# Effect of localized UV irradiation on the crystallinity and electrical properties of dip-coated polythiophene thin films

**DOI:** 10.1039/d0ra06339h

**Published:** 2020-09-15

**Authors:** So Young Park, Eun Hye Kwon, Yeong Don Park

**Affiliations:** a Department of Energy and Chemical Engineering, Incheon National University Incheon 22012 Republic of Korea ydpark@inu.ac.kr

## Abstract

Ensuring high performance in polymer devices requires conjugated polymers with interchain π–π stacking interactions *via* van der Waals forces, which can induce structural changes in the polymer thin film. Here, we present a systematic study of using simple localized UV irradiation to overcome the low crystallinity and poor charge carrier transport in dip-coated poly(3-hexylthiophene) (P3HT) thin films, which are consequences of the limited selection of solvents compatible with the dip-coating process. UV irradiation for only a few minutes effectively promoted P3HT chain self-assembly and association in the solution state. Brief UV irradiation of a P3HT solution led to well-ordered molecular structures in the resultant P3HT films dip-coated using a low boiling point solvent with rapid solvent evaporation. In addition, the position at which UV light was irradiated on the dip-coating solutions was varied, and the effects of the irradiation position and time on the crystallinity and electrical properties of the resultant P3HT thin films were investigated.

## Abstract 2

When the top part of the solution was irradiated with UV light, the dip-coated P3HT film showed enhanced crystallinity and electrical properties.

## Graphical abstract

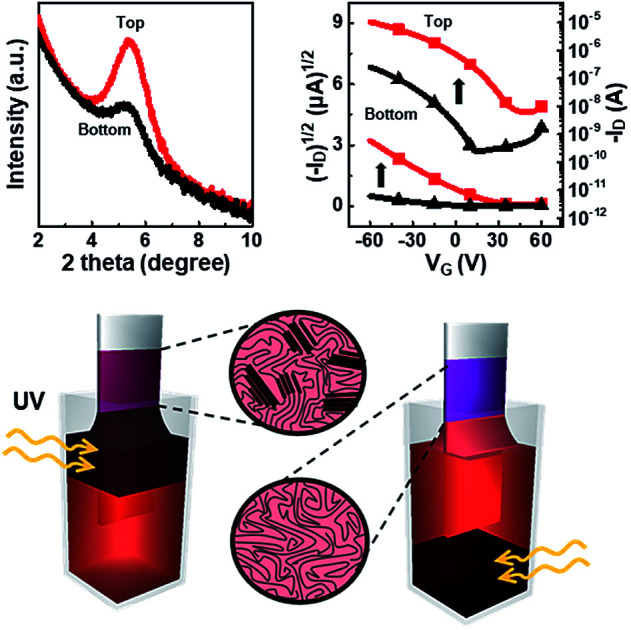

## 1. Introduction

Semiconducting polymers have the advantage of being manufactured at low cost *via* a simple solution-processing method.^1–4^ Among the methods used to form a polymer thin film, the dip-coating technique has numerous advantages, including the ability to form a uniform and large thin film with low roughness on various substrates.^5–7^ This method is widely used in academic studies and also in industrial production because it is suitable for continuous roll-to-roll processing.^8,9^ In the solution process, low boiling point solvents tend to form polymer thin films with low crystallinity because of the high evaporation rate of the solvent, resulting in films with poor charge carrier transport.^10^ Numerous studies have shown that high boiling point solvents produce highly ordered aggregates in polymer thin films because of the polymer chains have sufficient time for self-assembly.^11–13^ However, in the dip-coating process, high boiling point solvents produce inhomogeneous films with nonuniform thickness or dewetted regions because of the slow evaporation rate of the solvent; thus, the use of a solvent with a low boiling point is required.^14^

To ensure high performance of organic field-effect transistors (OFETs), interchain π–π stacking interactions of a polymer *via* van der Waals forces are needed; however, these interactions can lead to structural defect in the conjugated polymer film during film fabrication.^15–17^ Structural defects induced by polymer chain entanglements can limit charge carrier transport, resulting in poor device performance. In most inorganic materials, charge transport occurs as band transport because of high regularity of the atomic arrangement; by contrast, charge carrier transport in polymer molecules occurs *via* hopping transport between irregular arrangements *via* poor intermolecular coupling interactions.^18,19^ Device performance can be improved using various approaches to enhance the molecular order in active polymer layers; these approaches include nonsolvent addition,^20–22^ evaporation rate control,^10,23^ and post-treatments.^24,25^ UV irradiation of a conjugated polymer has recently been reported to induce the formation of ordered aggregates, demonstrating that UV irradiation expands the polymer chains in the absence of another post-treatment. Brief UV irradiation times have been shown to promote the formation of poly(3-hexylthiophene) (P3HT) ordered aggregates, leading to an increase in the number of nanofibrils in the resultant spin coated thin films, which in turn enhances charge carrier transport in the films.^26–28^

In the present study, we present a systematic study of using simple UV irradiation to overcome the low crystallinity and poor charge carrier transport in dip-coated P3HT thin films, which are consequences of the limited selection of solvents compatible with the dip-coating process. UV irradiation for only a few minutes induces crystal growth of P3HT in solution; from this perspective, a systematic and in-depth study of the crystallization kinetics of a P3HT conjugated polymer solution at the air–solution–substrate interfaces during a dip-coating process is presented. The processing conditions for enhancing the molecular order were optimized; in particular, the effects of UV irradiation position and irradiation time on the characteristics of P3HT thin films were investigated.

## 2. Experimental

### 2.1. Preparation of P3HT films and OFET devices

The P3HT with a weight-average molecular weight of 83 kDa and regioregularity = 89–92% was purchased from Rieke Metals as the semiconducting material. The OFETs were fabricated as top-contact bottom-gate type, and a highly n-doped Si wafer with a 3000 Å thick layer of SiO_2_ was used for the gate electrode and the insulator. Additionally, the SiO_2_ surface was spin coated with hexamethyldisilazane as an interlayer between the semiconducting layer and the dielectric layer. The P3HT was dissolved in chloroform at 50 °C to form a concentration of 5 mg mL^−1^. The prepared P3HT solution was cooled to room temperature before being irradiated with a UV-lamp (VILBER, VL-4.LC, 4 W) in a darkroom under ambient conditions. P3HT solutions were irradiated with UV light of 254 nm for various times (0–10 min). Either the whole P3HT solution or its upper or bottom regions was selectively irradiated with UV light for different exposure times. The substrate (*H* 2.5 cm × *L* 1 cm) was dipped into the UV irradiated P3HT solution for each experimental condition and was withdrawn at a constant rate of 5 mm s^−1^. The source and drain electrodes were fabricated by evaporating gold (∼40 nm thickness); these electrodes were used to measure the electrical performance of OFETs and were patterned using a shadow mask (channel length = 100 μm and channel width = 1000 μm). P3HT thin films for UV-vis spectrophotometric analysis were coated onto a transparent glass substrate using the aforementioned dip-coating method.

### 2.2. Characterization

UV-vis absorption spectra were collected using a UV-vis spectrophotometer (Lambda 365, PerkinElmer). The surface morphologies of the P3HT thin films dip-coated onto Si wafers were investigated by atomic force microscopy (AFM) (Multimode 8, Bruker) in tapping mode. Grazing-incidence X-ray diffraction (GIXD) profiles of the P3HT thin films were obtained using a high-resolution X-ray diffractometer (SmartLab, Rigaku). The characteristic current–voltage curves of the OFETs were recorded at room temperature under vacuum and in a dark environment using a semiconductor analyzer (Keithley 4200-SCS). The field-effect mobility in the P3HT films was calculated from the transfer curves in the saturation regime. A total of 40–50 devices were tested, and the measurement values related to their device performance under a given set of experimental conditions were averaged. The thicknesses of the P3HT films were measured with an ellipsometer (J. A. Woollam Co., Inc.).

## 3. Results and discussion

We chose P3HT, the most extensively studied conjugated polymer, as a representative polymer and investigated its crystallization and film solidification behavior; we selected chloroform as the main solvent to obtain a uniform and pinhole-free film. In the dip-coating process, the use chlorobenzene or dichlorobenzene, both which have a high boiling point, led to a nonuniform P3HT film with dewetted regions.^14^

**Fig. 1:**
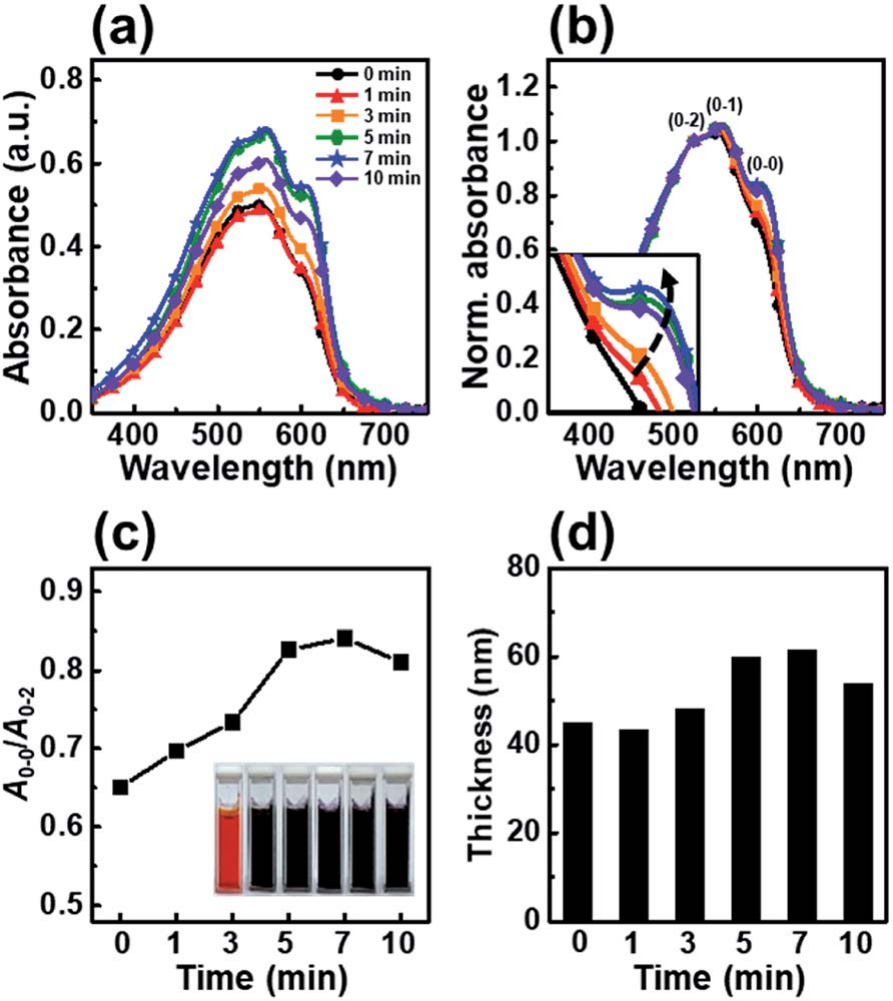
(a) UV-vis absorption spectra of dip-coated P3HT films using a solution irradiated with UV light for various times. (b) Normalized UV-vis absorption spectra of a dip-coated P3HT film using a solution irradiated with UV light for various times; the spectra are normalized to the *A*_0–2_ transition (*λ* = 522 nm). The inset shows a magnification of the *A*_0–0_ transition. (c) The ratio of the intensities of the *A*_0–0_ and *A*_0–2_ transitions in the absorption spectra of P3HT films. (d) Thickness of the P3HT films prepared with solutions irradiated for different times. The inset photos in (c) show the color change of the solutions irradiated with UV light for various times (0–10 min).

Fig. 1 shows the UV-vis absorption spectra of P3HT films dip-coated from solutions irradiated with UV light for irradiation times ranging from 0 to 10 min. The spectra of the P3HT thin films show three peaks corresponding to the *A*_0–0_, *A*_0–1_ and *A*_0–2_ absorption transitions. As reported previously, the spectra of the P3HT thin films showed *A*_0–2_ peaks at *λ* = 522 nm due to an intrachain π–π* transition and energy features associated with vibrionic bands at *λ* = 558 and 605 nm, corresponding to the first transition (*A*_0–1_) and second transition (*A*_0–0_), respectively.^29,30^ To precisely estimate the change in molecular order of the P3HT thin films, we normalized their UV-vis absorption spectra (Fig. 1b) and plotted *A*_0–0_/*A*_0–2_ (Fig. 1c), which represents the *A*_0–0_ absorbance intensities normalized to the *A*_0–2_ absorbance peak at 522 nm, as a function of the UV irradiation time. The spectrum of the pristine P3HT film shows low intensities of the *A*_0–0_ and *A*_0–1_ transitions because of the fast solvent evaporation rate; however, the intensities of the *A*_0–0_ and *A*_0–1_ transitions gradually increased with increasing UV irradiation time. This result is attributed to an increase in the number of ordered aggregates involved in the interaction between the interchain π–π stacking.^31^ The results indicate that the conformational structure of the P3HT main chains are partially changed under UV irradiation, which results in ordered aggregates. The color of the P3HT solution changes from orange to dark-brown with increasing UV irradiation time, which indicates that the number of well-ordered P3HT molecules increased (inset in Fig. 1c).^32^ When the UV irradiation time was longer than 7 min, the intensities of both the *A*_0–1_ and *A*_0–0_ transitions decreased, indicating that the molecular order in the dip-coated P3HT film was reduced.^33^ Increasing the UV-vis absorbance intensity led to an increase in film thickness, as determined using the Beer–Lambert law. With increasing UV irradiation time, the film thickness gradually increased, reaching a maximum thickness at 7 min (Fig. 1d). However, when the UV irradiation time exceeded 10 min, the thickness slightly decreased. The film thickness trends observed by ellipsometry well match those based on the UV-vis absorbance peak intensity.

**Fig. 2:**
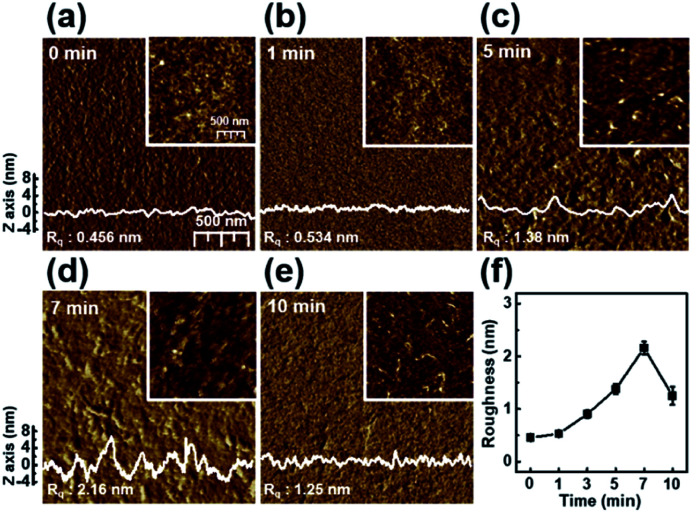
Tapping-mode AFM phase images of the P3HT films prepared with solutions irradiated with UV light for various times: (a) 0, (b) 1, (c) 5, (d) 7, and (e) 10 min. The insets show height images of the films. The surface profiles were extracted from the height image. (f) The roughness of the P3HT films as a function of UV irradiation time.

Fig. 2 shows the nanoscale morphology and surface roughness through AFM phase images of P3HT films dip-coated from solutions irradiated with UV light for various times. With increasing UV irradiation time, the number of ordered aggregates and roughness of the P3HT films increased because of association of the polymer chains. These morphological results confirm that UV irradiation transformed the P3HT chain structure, resulting in ordered aggregates.^34^ In the case of the UV irradiation time at 10 min, the roughness decreased because the molecular order of the film deteriorated.

**Fig. 3:**
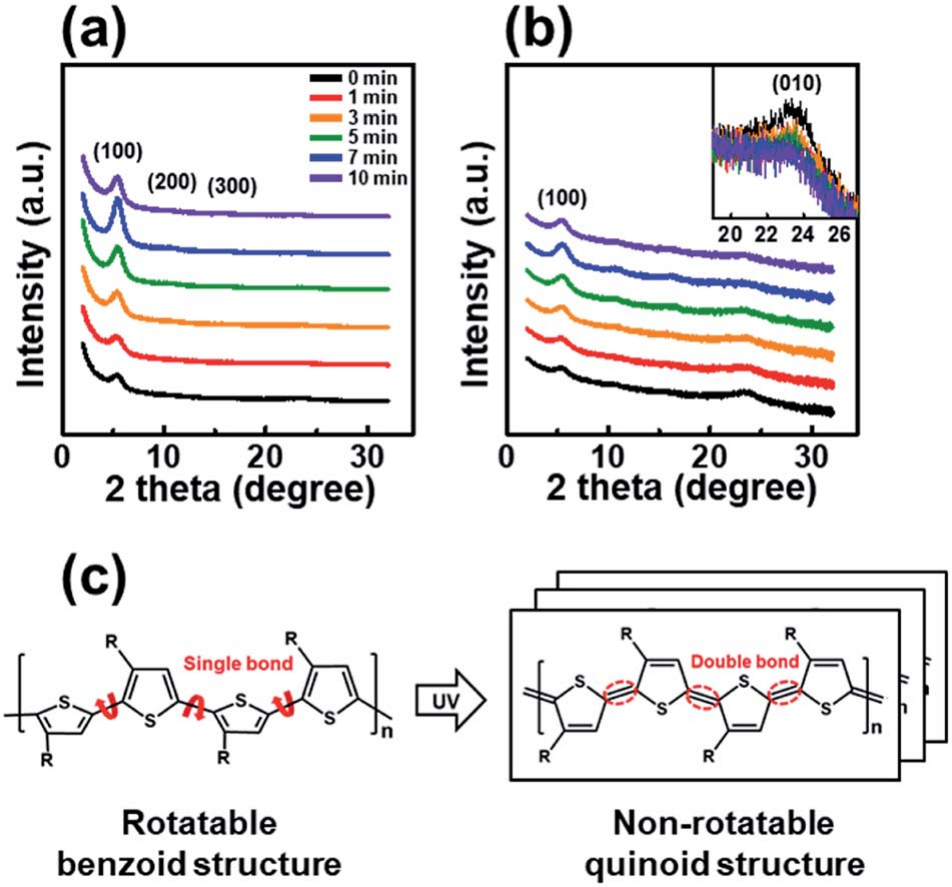
The out-of-plane GIXD patterns of the P3HT films prepared with solutions irradiated with UV light for different times, as plotted on (a) linear and (b) logarithmic axes. The inset shows a magnification of the (010) peak. (c) Structural change of the P3HT chain from benzoidal to quinoidal in the P3HT molecular structure under UV irradiation.

The trend observed in the AFM images is consistent with the data obtained from the X-ray diffraction analysis shown in Fig. 3. The out-of-plane GIXD data were obtained from a grazing-incidence measurement of P3HT films dip-coated from solutions subjected to UV irradiation for various times. The (100) peak due to the lamellar layered structure appears at 5.42°, and the (010) peak due to π–π interchain stacking in the P3HT film appears at 23.34°. With increasing UV irradiation time, the intensity of the (100) peak associated with P3HT lamellar stacking gradually increases along the crystallographic direction perpendicular to the backbone. This improvement is due to the increase in the amount of crystals.^35^ Furthermore, in the case of a pristine film, the out-of-plane (010) diffraction peaks associated with the π–π interconnect stacking are relatively strong (Fig. 3b), indicating a face-on orientation. As the UV irradiation time increases, the (010) diffraction peak gradually decreases in intensity, indicating a reorganization toward more desirable edge-on orientation relative to the substrate. The edge-on orientation is beneficial for OFET performance because it promotes charge carrier transport in the lateral direction.^36^ These results indicate that UV irradiation of the P3HT solution results in improved crystallinity and a preferred chain orientation for charge carrier transport in an OFET. The P3HT aggregates grown for 7 min exhibited the highest crystallinity as well as the preferred orientation, suggesting the existence of an optimum UV exposure time. Fig. 3c shows the mechanism of P3HT chain transformation under UV irradiation. The effect of UV irradiation can be partially explained through a change in the conformational structure of the P3HT backbone. Upon UV irradiation, the P3HT molecular chain changes from a benzoid conformation to a quinoid conformation.^27^ The benzoid structure is predominantly a random-coil form because of the presence of a rotatable single-bond, whereas the quinoid structure is predominantly a linear or extended-coil form with a rigid backbone because of the double-bond character of the P3HT inter-ring bond. The quinoid structure, which has a higher degree of coplanarity, induces neighboring π–π interactions, which results in an ordered aggregate of P3HT molecules.^37^

**Fig. 4:**
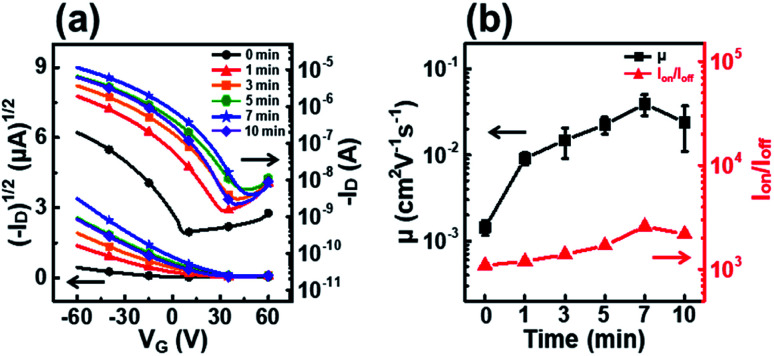
(a) Plots of the drain current *versus* the gate voltage at a fixed drain voltage (*V*_D_ = −60 V) on both linear (left axis) and log(right axis) scales for P3HT films prepared with solutions irradiated with UV light for different times. (b) Average field-effect mobilities (left axis) and on–off ratio (right axis) of the OFETs fabricated using P3HT films irradiated with UV light for different times.

Fig. 4 shows the transfer curves, field-effect mobility, and on–off ratio of P3HT films dip-coated from solutions irradiated with UV light for various times. The field-effect mobility gradually increased with increasing UV irradiation time, and the best field-effect mobility of 3.9 × 10^−2^ cm^2^ V^−1^ s^−1^ corresponded to an irradiation time of 7 min (Table 1). This improvement in the field-effect mobility can be explained by an increase in crystallinity and favorable molecular orientation in the P3HT films, as confirmed by UV-vis and GIXD analyses.^38^ However, the field-effect mobility does not increase when the irradiation time is longer than 7 min because severe P3HT aggregation occurs in the dip-coating processed film. The decrease in mobility of films prepared from solutions irradiated for longer than 7 min is explained by the deterioration of the molecular order due to P3HT precipitates hundreds of micrometers in size as a result of the excessively long UV irradiation (data not shown). This trend in device performance is exactly consistent with the UV-vis absorption spectra and out-of-plane GIXD patterns of the P3HT films.

**Table 1:** Summary of the electrical properties of P3HT films prepared using solutions irradiated with UV light for different times

Time (min)	*μ* (cm^2^ V^−1^ s^−1^) (×10^−2^)	*I*_on_/*I*_off_ (×10^3^)	*V*_th_ (V)
0	0.18	1.1	7.0
1	0.91	1.2	32
3	1.5	1.4	39
5	2.3	1.7	45
7	3.9	2.6	48
10	1.9	2.2	39

**Fig. 5:**
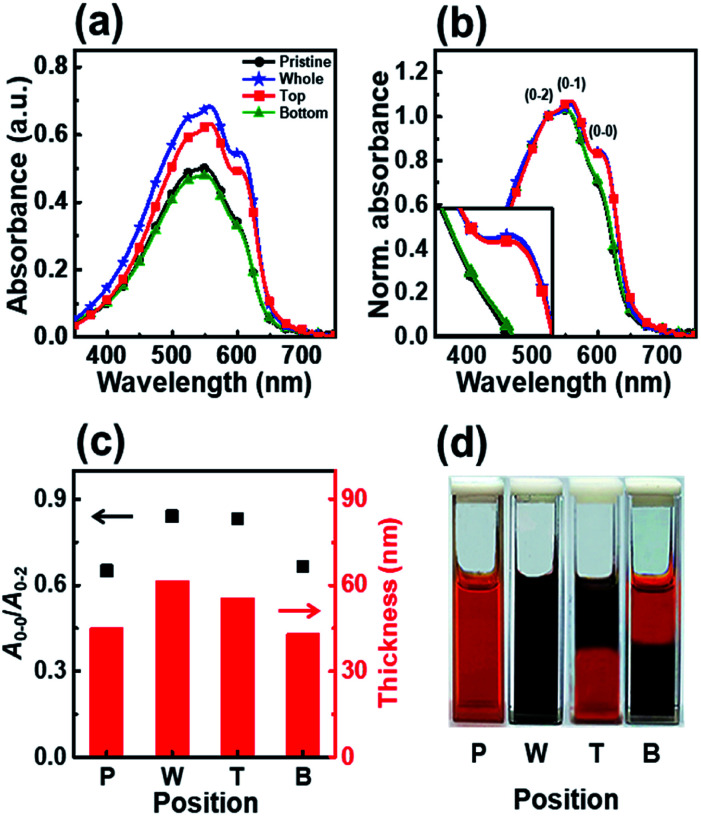
(a) UV-vis absorption spectra of dip-coated P3HT film prepared using solutions irradiated with UV light at various positions. (b) Normalized UV-vis absorption spectra of P3HT films dip-coated using solutions irradiated with UV light at different positions; the spectra were normalized to the *A*_0–2_ transition (*λ* = 522 nm). (c) The ratio of the intensities of the *A*_0–0_ and *A*_0–2_ transitions (left axis) and the thickness (right axis) of P3HT films prepared with solutions irradiated at different positions. (d) Photograph showing the color change of the P3HT solution according to the UV irradiation position.

To gain further insight into the effects of UV irradiation, we studied the effect of irradiation position on the molecular order of the P3HT molecules. We monitored the aggregation of P3HT molecules in the upper or lower regions of the dip-coating solution as a function of the irradiation position and determined the effect of the irradiation position on the molecular structure of the dip-coated films. We tested two different positions, top and bottom, as the UV irradiation region with an identical irradiation time of 7 min. Fig. 5 shows the UV-vis absorption spectra of the P3HT films dip-coated in solutions with different UV irradiation positions. The intensities of the *A*_0–1_ and *A*_0–0_ transitions in the P3HT film prepared with irradiation of the top part of the solution (film T) showed a high intensity similar to that of the intensities in the spectrum of the P3HT film prepared using a solution whose whole volume was irradiated (film W) (Fig. 5b). However, when the bottom part of the solution was irradiated, the absorption spectrum of the resultant P3HT film (film B) showed *A*_0–1_ and *A*_0–0_ transition intensities similar to those in the spectrum of the pristine P3HT film (film P).

**Scheme 1:**
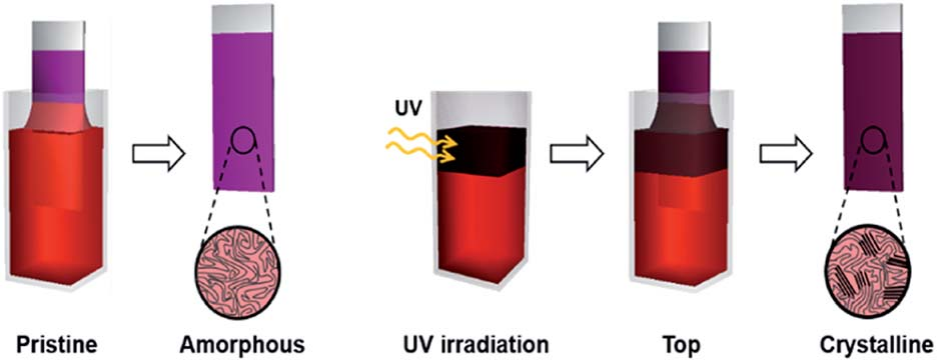
Schematic showing experimental process and improved crystallinity of the P3HT film formed with UV irradiation of the top region during the dip-coating process (film T).

The enhanced crystallinity of film T can be explained by the mechanism of the dip-coating process. In general, the dip-coating process is separated into three stages: immersion, dwell period, and withdrawal.^39^ The substrate is dipped into a solution at a controlled speed. During the withdrawal process, the solution is pulled to the substrate through surface tension and is dried at the contact line, where it is coated onto the substrate. Solidification of polymer semiconductors during dip-coating is an evaporation-induced process that occurs at the contact line, where air–solution–substrate interfaces exist. Therefore, the characteristic of the top area in a polymer solution determines the main properties of the resulting film during a dip-coating process.^5–7^ Film T has similar crystallinity and thickness as film W because the number of ordered aggregates in the top area of the solution increased, which involves the interaction between interchain π–π stacking layers under UV irradiation (Scheme 1). During the dip-coating process, the polymer chain structure in the top region of the solution strongly affected the resulting film formation and crystallization structure. By contrast, film B has a similar molecular order as film P because UV irradiation of the lower part of the solution did not affect the surface property of the P3HT solution. These results strongly suggest that the growth of ordered aggregates in the film state was governed not only by the UV exposure time but also by the region irradiated during the dip-coating process. The thickness of films W and T was 61 nm and 56 nm, respectively, which are slightly thicker than film P because of aggregation of the crystals. The color of the P3HT solution locally changes from orange to dark-brown (Fig. 5d) depending on the UV irradiation position.^32^

**Fig. 6:**
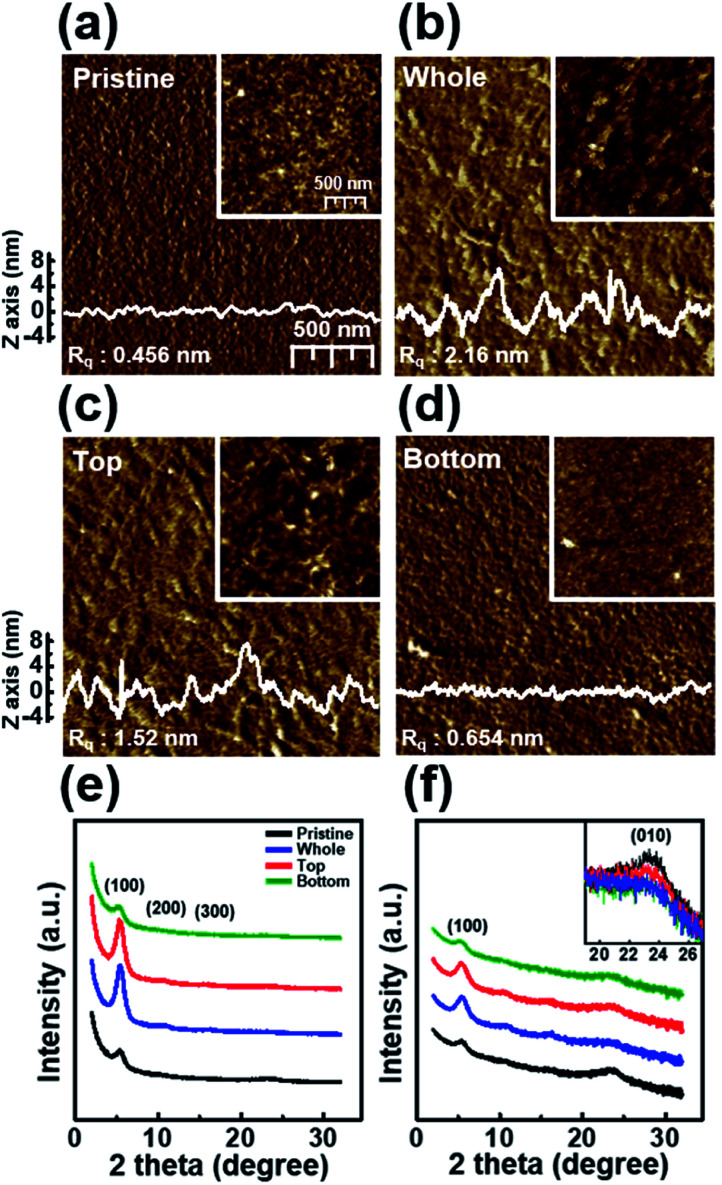
Tapping-mode AFM phase images of (a) a pristine P3HT film (P) and films prepared with solutions irradiated with UV light at different positions: (b) whole (W), (c) top (T), and (d) bottom (B). The surface profiles were extracted from the height image. The insets show height images of the P3HT films. The out-of-plane GIXD patterns of the P, W, T, and B P3HT films plotted on (e) linear and (f) logarithmic axes. The inset shows a magnification of the (010) peak.

Fig. 6 shows the nanoscale morphology, as characterized using AFM images and the GIXD pattern of a dip-coated P3HT film depending on the UV irradiation position. The AFM images clearly reveal that the films W and T exhibit increased roughness because of increased ordering of their structure,^34^ whereas film B exhibits a featureless and homogeneous morphology. In addition, to analyze the crystallinity according to the UV irradiation position, the out-of-plane GIXD data were obtained from grazing-incidence measurements of P3HT films dip-coated from solutions with different UV irradiation positions. The intensity of the (100) peaks in the patterns of films W and T increased along the crystallographic direction perpendicular to the skeleton, whereas the intensities of the (010) diffraction peaks decreased. These results are in good agreement with the UV-vis spectrum and AFM results.

**Fig. 7:**
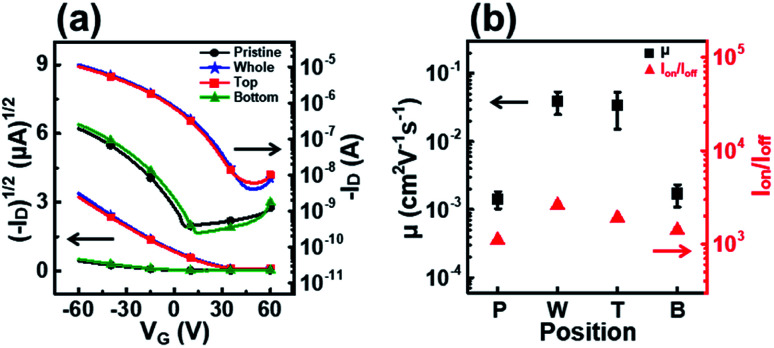
(a) Plots of the drain current *versus* the gate voltage at a fixed drain voltage (*V*_D_ = −60 V) on both linear (left axis) and logarithmic (right axis) scales for OFETs fabricated using P3HT films prepared from solutions irradiated with UV light at different positions. (b) Average field-effect mobilities (left axis) and on–off ratio (right axis) of the OFETs fabricated using P3HT films prepared from solutions irradiated with UV light at different positions.

Fig. 7 shows the transfer curves, field-effect mobility, and on–off ratio of OFETs fabricated using P3HT films dip-coated with solutions UV irradiated at different positions. The devices fabricated with films T and W showed high field-effect mobilities of 3.4 × 10^−2^ and 3.9 × 10^−2^ cm^2^ V^−1^ s^−1^, respectively (Table 2). By contrast, the film B device exhibited similar charge transport properties as the film P device. The results also show that the UV irradiation position during the dip-coating process strongly affects the molecular order and electrical properties of the resultant films.

**Table 2:** Summary of the electrical properties of P3HT films prepared from solutions irradiated with UV light at different positions

Position	*μ* (cm^2^ V^−1^ s^−1^) (×10^−2^)	*I*_on_/*I*_off_ (×10^3^)	*V*_th_ (V)
Pristine	0.18	1.1	7.0
Whole	3.9	2.6	48
Top	3.4	1.9	48
Bottom	0.19	1.4	14

## 4. Conclusions

A systematic study of simple UV irradiation exposure for overcoming the low crystallinity and low charge carrier transport in P3HT thin films during dip-coating process was presented. UV irradiation transformed the polymer chain conformation from benzoid to quinoid within only a few minutes and promoted polymer chain aggregation. The formation of polymer aggregates *via* favorable interchain π–π stacking in solution improved the molecular order and charge transport of that resultant dip-coated polymer films. The P3HT aggregates grown for 7 min exhibited the highest crystallinity as well as the preferred orientation, suggesting the existence of an optimum UV exposure time. When the UV irradiation time was longer than 7 min, the molecular order in the dip-coated P3HT film was reduced due to P3HT precipitates hundreds of micrometers in size. Furthermore, when UV light was irradiated to the top part of the solution, the dip-coated P3HT film showed improved crystallinity and high field-effect mobilities of 3.4 × 10^−2^ cm^2^ V^−1^ s^−1^. These results elucidate the polymer-solution crystallization kinetics at the air–solution–substrate interfaces. This facile localized solution crystallization approach represents an effective alternative for the large-scale fabrication and high-throughput processing of highly crystalline polymer films because of the easy processing conditions compared to with those in additive-induced solution crystallization or other post-treatment methods. Our research provides practical guidelines for fabrication of crystalline polymer films *via* the dip-coating process.

## Conflicts of interest

There are no conflicts to declare.

## Supplementary Material
